# Machine learning in the diagnosis of asthma phenotypes during coronavirus disease 2019 pandemic

**DOI:** 10.1002/clt2.12201

**Published:** 2022-10-19

**Authors:** Agnieszka Gawlewicz‐Mroczka, Adam Pytlewski, Natalia Celejewska‐Wójcik, Adam Ćmiel, Anna Gielicz, Marek Sanak, Lucyna Mastalerz

**Affiliations:** ^1^ Department of Internal Medicine Jagiellonian University Medical College Krakow Poland; ^2^ University Hospital Krakow Poland; ^3^ Department of Applied Mathematics AGH University of Science and Technology Krakow Poland

**Keywords:** COVID‐19 pandemic, machine learning, nonsteroidal anti‐inflammatory drug (NSAID)–exacerbated respiratory disease (NERD), nonsteroidal anti‐inflammatory drug tolerant asthma (NTA), oral aspirin challenge

## Abstract

**Background:**

During the coronavirus disease 2019 (COVID‐19) pandemic, it has become a pressing need to be able to diagnose aspirin hypersensitivity in patients with asthma without the need to use oral aspirin challenge (OAC) testing. OAC is time consuming and is associated with the risk of severe hypersensitive reactions. In this study, we sought to investigate whether machine learning (ML) based on some clinical and laboratory procedures performed during the pandemic might be used for discriminating between patients with aspirin hypersensitivity and those with aspirin‐tolerant asthma.

**Methods:**

We used a prospective database of 135 patients with non‐steroidal anti‐inflammatory drug (NSAID)–exacerbated respiratory disease (NERD) and 81 NSAID‐tolerant (NTA) patients with asthma who underwent OAC. Clinical characteristics, inflammatory phenotypes based on sputum cells, as well as eicosanoid levels in induced sputum supernatant and urine were extracted for the purpose of applying ML techniques.

**Results:**

The overall best ML model, neural network (NN), trained on a set of best features, achieved a sensitivity of 95% and a specificity of 76% for diagnosing NERD. The 3 promising models (i.e., multiple logistic regression, support vector machine, and NN) trained on a set of easy‐to‐obtain features including only clinical characteristics and laboratory data achieved a sensitivity of 97% and a specificity of 67%.

**Conclusions:**

ML techniques are becoming a promising tool for discriminating between patients with NERD and NTA. The models are easy to use, safe, and achieve very good results, which is particularly important during the COVID‐19 pandemic.

## 1 BACKGROUND

Asthma is a heterogeneous disease with various phenotypes. Asthma with aspirin hypersensitivity is a specific phenotype accompanied by chronic rhinosinusitis with nasal polyposis (CRSwNP) and characterized by a severe course. Recently, it has been termed “nonsteroidal anti‐inflammatory drug (NSAID)‐exacerbated respiratory disease (NERD)”.^1^ Several decades ago, it was observed that hypersensitivity to NSAIDs manifests with asthma accompanied by CRSwNP. This is how aspirin‐intolerant asthma was distinguished. It was later renamed as aspirin‐exacerbated respiratory disease, which pointed to the underlying inflammatory process affecting the upper and lower airways.^1^

The pathogenesis of NERD is associated with several abnormalities related to the cyclooxygenase (COX) and lipoxygenase pathways of arachidonic acid metabolism in the upper and lower airway mucosa. A reduced expression of COX_**2**_ mRNA leading to a lower generation of PGE_**2**_ by nasal polyps,^2^ nasal polyp epithelial cells,^2^ and bronchial fibroblasts was reported.^2^ This, together with a reduced expression of prostaglandin EP2 receptors, could result in impaired anti‐inflammatory response.^3^, ^4^ An increased generation of cysteinyl leukotrienes as well as overexpression of enzymes involved in leukotriene production (5‐lipoxygenase and leukotriene C4 synthase), together with an increased expression of leukotriene type 1 receptors in the nasal mucosa of patients with NERD, may result in local hyperresponsiveness to leukotrienes.^1^

With a sensitivity of 89% and a specificity of 93%, oral aspirin challenge (OAC) remains the gold standard for diagnosing aspirin hypersensitivity.^5^ Recently, the coronavirus disease 2019 (COVID‐19) pandemic has strongly limited OAC use, because aerosol‐producing medical procedures have been forbidden. Moreover, OAC is time consuming, requires experienced personnel, may cause severe systemic reaction, and cannot be performed in patients with impaired lung function (forced expiratory volume in the first second [FEV_**1**_] < 70%) or previous anaphylactic shock caused by NSAIDs. Inhaled or intranasal aspirin challenge tests can be performed instead of OAC, but they are limited by a need for specialist equipment and well‐trained staff.^5^ Thus, there is now a pressing need to develop simple tools for a differential diagnosis of aspirin‐tolerant and aspirin‐sensitive asthma based on clinical parameters and medical history data rather than OAC testing.

Currently, there are no reliable in vitro diagnostic tests for routine use in patients with NERD. Recently, artificial intelligence has emerged as an increasingly useful tool in different medical fields. It was reported to facilitate the diagnosis of respiratory diseases, such as chronic obstructive pulmonary disease and NERD.^6^, ^7^ We previously described the possible role of the artificial neural network (NN) in diagnosing NERD using as many data as possible, including induced sputum (IS) and spirometry parameters.^7^

In this study, we aimed to investigate the usefulness of machine learning (ML) techniques in discriminating between patients with aspirin‐sensitive and those with aspirin‐tolerant asthma. The goal was to use easily obtainable data from medical history as well as clinical and laboratory data, while omitting procedures that were not allowed during the COVID‐19 pandemic (due to the risk of virus transmission), such as aspirin challenge, IS testing, and spirometry.

## 2 METHODS

### 2.1 Study group

In this study, we used a prospective database of 135 patients with NERD and 81 patients with NSAID‐tolerant asthma (NTA), who were recruited from among patients with asthma treated at the Andrzej Szczeklik Department of Internal Medicine, Jagiellonian University Medical College, Krakow, Poland.

Of the 135 patients, 71% reported previous adverse reactions after NSAID use. However, in the remaining 29% of patients, the precise history of NSAID use could not be determined. Some patients were ineligible for long‐term NSAID use due to CRSwNP and the risk of NSAID hypersensitivity.

Hypersensitivity to NSAIDs was confirmed by OAC according to the European Academy of Allergy and Clinical Immunology guidelines.^5^ Asthma severity was assessed based on the 2021 Global Initiative for Asthma report^8^ and the presence of CRSwNP confirmed by ear, nose, and throat examination and sinus computed tomography.

The clinical characteristics of patients were collected between 2014 and 2021 (Table 1). Patients were participants of our previous studies on aspirin hypersensitivity (see Acknowledgments). Biological samples including blood, IS, and urine were obtained at the following time points: (a) 1 day before bronchial aspirin challenge^9^; (b) 1.5 h before diagnostic OAC^10^; (c) 1 day before OAC performed because of aspirin desensitization^11^; and (d) 1 day before OAC (unpublished data). Asthma control was assessed using the Asthma Control Test (ACT). Subjects remained clinically stable, and the FEV_**1**_ was ≥70% of predicted value on the day of aspirin challenge. None of the participants experienced any asthma exacerbation or respiratory tract infection during the 6 weeks preceding the study. Moreover, none of the patients with asthma had been treated with leukotriene modifiers 6 weeks prior to the study or with other medications except inhaled corticosteroids (ICSs), small doses of oral corticosteroids (OCSs; ≤10 mg of prednisolone or equivalent), and long‐acting **β**_**2**_‐agonists. Patients with a history of biologic treatment were also excluded. The primary outcome was the diagnosis of NERD. For the purpose of this study, we considered patient's clinical data (sex, age at asthma onset, body mass index, ACT score, asthma severity, ICS and OCS treatment, presence of CRSwNP, history of sinonasal surgery), FEV_**1**_ value, and the results of skin prick tests to aeroallergens. Laboratory tests included blood eosinophil count, total serum immunoglobulin E levels, inflammatory phenotypes based on different cut‐off levels of cell percentage, concentrations of prostaglandins PGD_**2**_ and PGE_**2**_ as well as leukotrienes LTE_**4**_ and LTD_**4**_ in IS supernatant (ISS), and urinary LTE_**4**_ levels.

**TABLE 1:** Characteristics of the study groups

Feature	NERD (*n* = 135)	NTA (*n* = 81)	*p*‐value
Age (years)	47.0 ± 11.9	47.5 ± 14.2	0.809
Sex (female/male)	97/38	43/38	0.005
BMI (kg/m^2^)	27.0 ± 5.1	26.7 ± 4.4	0.608
Asthmaonset (years)	34.5 ± 12.7	33.4 ± 17.2	0.621
Asthmaduration (years)	12.7 ± 8.9	14.2 ± 11.8	0.387
Asthmacontrol (good/mid/bad)	89/28/18	65/10/6	0.088
Asthmaseverity (mild/moderate/severe)	19/17/99	29/9/43	0.001
Pastsinonasalsurgeries (yes/no)	122/13	40/41	<0.001
CRSwNP (yes/no)	135/0	43/38	<0.001
ICS (yes/no)	122/13	62/19	0.009
OCS (yes/no)	8/127	7/74	0.432
Pricktests (positive/negative)	49/86	52/29	<0.001
ACTscore (points)	20.8 ± 4.4	22.0 ± 3.9	0.014
BaselineFEV₁ (%)	89.9 ± 15.5	95.2 ± 16.0	0.019
DoseofICS (µg/d fluticasone eq)	664.5 ± 457.3	537.1 ± 508.2	0.010
Bloodeosinophils (mm³)	415.6 ± 306.2	377.7 ± 335.6	0.092
TotalserumIgE (IU/ml)	188.0 ± 250.2	332.4 ± 954.6	0.485
ISneutrophils (%)	39.8 ± 22.1	46.9 ± 22.4	0.024
ISeosinophils (%)	9.7 ± 13.8	4.0 ± 8.6	<0.001
ISSPGD₂	65.7 ± 76.2	57.7 ± 169.9	0.006
ISSPGE₂	87.3 ± 100.5	85.1 ± 100.6	0.406
ISSLTD₄	108.1 ± 353.3	66.4 ± 102.0	0.248
ISSLTE₄	114.7 ± 166.5	49.0 ± 87.1	<0.001
UrinaryLTE₄ (pg/mg creatinine)	2743.1 ± 5796.7	8023.7 ± 64,517.3	<0.001
ISphenotypeneutrophilic (yes/no)^a^	19/116	20/61	0.050
ISphenotypeeosinophilic (yes/no)^a^	61/74	17/64	<0.001
ISphenotypepaucigranulocytic (yes/no)^a^	47/88	40/41	0.035
ISphenotypemixed (yes/no)^a^	8/127	4/77	1.000
ISphenotypeneutrophilic (yes/no)^b^	14/121	17/64	0.031
ISphenotypeeosinophilic (yes/no)^b^	70/65	24/57	0.001
ISphenotypepaucigranulocytic (yes/no)^b^	37/98	33/48	0.043
ISphenotypemixed (yes/no)^b^	14/121	7/74	0.678
ISphenotypeeosinophilic (yes/no)^c^	63/72	18/63	<0.001
ISphenotypenoneosinophilic (yes/no)^c^	66/69	60/21	<0.001
ISphenotypemixed (yes/no)^c^	6/129	3/78	1.000

*Note*: Data are presented as mean ± SD or number of patients.

Abbreviations: ACT, Asthma Control Test; BMI, body mass index; CRSwNP, chronic rhinosinusitis with nasal polyposis; FEV₁, forced expiratory volume in the first second; ICS, inhaled corticosteroids; IgE, immunoglobulin E; IS, inducted sputum; ISS, induced sputum supernatant; LTD₄, leukotriene D₄; LTE₄, leukotriene E₄; NERD, nonsteroidal anti‐inflammatory drug–exacerbated respiratory disease; NTA, nonsteroidal anti‐inflammatory drug–tolerant asthma; OCS, oral corticosteroids; PGD₂, prostaglandin D₂; PGE₂, prostaglandin E₂.

a, b, c There are several accepted thresholds when defining IS phenotypes.

^a^ Threshold of 3% for eosinophiles and 60% for neutrophiles, 4 phenotypes.

^b^ Threshold of 2% for eosinophiles and 60% for neutrophiles, 4 phenotypes.

^c^ Threshold of 3% for eosinophiles and 64% for neutrophiles, 3 phenotypes.

Each of the studies providing data for the current research was approved by Jagiellonian University Ethics Committee, and written informed consent was obtained from all study participants. The study was conducted in accordance with the Declaration of Helsinki.

### 2.2 Data collection

IS was obtained from all study participants before the OAC test, as described above.^9^, ^10^, ^11^ Samples were collected according to the European Respiratory Society recommendations.^12^ The material was processed to obtain cytospin slides for a differential cell count and a supernatant for eicosanoid evaluation. Data from the IS differential cell count were divided into foue cell phenotypes using different cut‐off values for eosinophils (2%^13^ and 3%^11^). Based on another publication, three cell phenotypes were distinguished.^14^

The levels of eicosanoids in ISS were measured by gas chromatography/mass spectrometry for PGD_**2**_ and PGE_**2**_ and by high‐performance liquid chromatography/tandem mass spectrometry for LTE_**4**_ and LTD_**4**_. Analytical details were described elsewhere.^9^

Urinary LTE_**4**_ levels were assessed with an enzyme‐linked immunosorbent assay (Cayman Chemical Co.). The results were recalculated in picograms per milligram of creatinine.

### 2.3 Methodology of machine learning

#### 2.3.1 Study design and workflow

Detailed characteristics of patients with a comparison between the NERD and NTA groups are presented in Table 1. In the database, information on urinary LTE**₄** levels was missing for nine patients; on LTE**₄** and LTD**₄** levels in ISS, for six patients; and on PGE**₂** and PGD**₂** levels in ISS, for two patients. Those values were imputed using the K‐Nearest Neighbors method (with K set to 5). Continuous features were normalized using the following formula:

$$normalized\;feature=\frac{feature-mean\;of\;the\;feature}{maximum\;of\;the\;feature-minimum\;of\;the\;feature}.$$

A flowchart of the consecutive steps, from obtaining the database of patients to the final evaluation of the best models, is presented in Figure S1 (part of the supplementary materials). We randomly split a database of 216 patients into two parts: the “training + validation” set with 156 patients (including 96 NERD patients) and the “test” set with 60 patients (including 39 NERD patients). We prepared three different subsets of features. Basically, the “all features” set contained all the possible features collected before the pandemic, including spirometry and IS testing parameters. The “best” set contained the best possible features that were chosen using the L‐1–based feature selection technique. A logistic regression with the L‐1 penalty was created. The L‐1 penalty has a property of making the model's coefficients sparse, and unimportant features are assigned a coefficient value of 0. Such features are considered unimportant and are eliminated. The “easy‐to‐obtain” set contained easily obtainable features, which were manually chosen by the authors. Those features were considered to be easily accessible during the pandemic when other tests were unavailable. The list of features for each set is presented in Table 2. We checked seven types of algorithms: decision tree, random forest, eXtreme Gradient Boosting (XGBoost), multiple logistic regression (MLR), support vector machine (SVM), NN, and TabNet.^15^ The best hyperparameter settings of those models were chosen with a 5‐fold cross‐validation, which is a way to choose the best models. The training + validation set was divided into five parts. The model was trained on the first four parts, and the final part remained for evaluation. This process was repeated five times, and each time a different one‐fifth part of the training + validation set was used for evaluation. The mean of five different evaluation results was considered when choosing the best hyperparameter settings for an algorithm. Next, the best model was retrained on the entire training + validation set and assessed on the test set.

**TABLE 2:** Features included in each dataset

Features names	Dataset with all features	Dataset with best features	Dataset with easy‐to‐obtain features
Age	*✓*	✗	*✓*
Asthmaonset	*✓*	✗	*✓*
Asthmaduration	*✓*	✗	*✓*
BMI	*✓*	✗	*✓*
ACTscore	*✓*	✗	*✓*
Bloodeosinophils	*✓*	✗	✗
TotalserumIgE	*✓*	✗	✗
BaselineFEV₁	*✓*	*✓*	✗
ICSdose	*✓*	✗	*✓*
ISneutrophils	*✓*	*✓*	✗
ISeosinophils	*✓*	✗	✗
ISSPGD₂	*✓*	✗	✗
ISSPGE₂	*✓*	✗	✗
ISSLTD₄	*✓*	✗	✗
ISSLTE₄	*✓*	✗	✗
UrinaryLTE₄	*✓*	✗	✗
Sex	*✓*	*✓*	*✓*
Previoussinonasalsurgeries	*✓*	✗	*✓*
CRSwNP	*✓*	*✓*	*✓*
ICS	*✓*	*✓*	*✓*
OCS	*✓*	✗	*✓*
Skinpricktests	*✓*	*✓*	*✓*
Asthmacontrol	*✓*	*✓*	*✓*
Asthmaseverity	*✓*	✗	*✓*
ISphenotypeneutrophilic ^a^	*✓*	✗	✗
ISphenotypeeosinophilic ^a^	*✓*	✗	✗
ISphenotypepaucigranulocytic ^a^	*✓*	✗	✗
ISphenotypemixed ^a^	*✓*	✗	✗
ISphenotypeneutrophilic ^b^	*✓*	*✓*	✗
ISphenotypeeosinophilic ^b^	*✓*	✗	✗
ISphenotypepaucigranulocytic ^b^	*✓*	✗	✗
ISphenotypemixed ^b^	*✓*	✗	✗
ISphenotypeeosinophilic ^c^	*✓*	✗	✗
ISphenotypenoneosinophilic ^c^	*✓*	*✓*	✗
ISphenotypemixed ^c^	*✓*	✗	✗

Abbreviations: ACT, Asthma Control Test; BMI, body mass index; CRSwNP, chronic rhinosinusitis with nasal polyposis; FEV₁, forced expiratory volume in the first second; ICS, inhaled corticosteroid; IgE, immunoglobulin E; IS, inducted sputum; ISS, induced sputum supernatant; LTD₄, leukotriene D₄; LTE₄, leukotriene E₄; OCS, oral corticosteroids; PGD₂, prostaglandin D₂; PGE₂, prostaglandin E₂.

a, b, c There are several accepted thresholds when defining IS phenotypes.

^a^ 3% threshold for eosinophiles and 60% for neutrophiles, 4 phenotypes.

^b^ 2% threshold for eosinophiles and 60% for neutrophiles, 4 phenotypes.

^c^ 3% threshold for eosinophiles and 64% for neutrophiles, 3 phenotypes.

#### 2.3.2 Algorithms

Given input features of the patient, an ML algorithm was supposed to predict an output label, which could be either NERD or NTA. There are various algorithms that can perform such a classification task, and they are controlled by different hyperparameters. Hyperparameters are settings that can be manually influenced by a person. Parameters, on the other hand, are learned by the algorithm itself. An example hyperparameter could be the number of neurons in each layer of an NN. Hyperparameter tuning is done with cross‐validation, and it allows to pick the best settings for a model. Detailed descriptions of the ML algorithms used in this study can be found in the supplementary materials.

#### 2.3.3 Model evaluation and statistical analysis

We used accuracy (correctly classified examples/total number of examples) as our evaluation metric for choosing the best hyperparameters for each of the seven algorithms. A model with the highest accuracy was considered the best. Each type of an algorithm in our study had its best hyperparameters chosen via 5‐fold cross‐validation. Models with the highest validation score were reported. For the best models, we assessed the following additional metrics: diagnostic accuracy (which takes into account the prevalence of NERD in asthma population—7%),^16^ sensitivity, specificity, and the area under the receiver operating characteristic curve (AUC) with 95% confidence intervals (CIs), which were provided in brackets. The Shapley Additive exPlanations (SHAP) analysis was performed for the best models to explain how they are predicting an output.

For statistical analysis, in the case of a continuous feature with normal distribution, we used the Student's *t*‐test for mean comparison. If the distribution was skewed, the Mann‐Whitney *U* test was used. For a categorical feature, we created a contingency table and compared the data using the **χ** ^**2**^ test if the size of every sample was at least 5. Otherwise, the Fisher's exact test was used. A *p*‐value  <0.05 was considered significant. All calculations, model training, and visualizations were done using Python 3.7.9 and the following libraries: Pandas 1.1.5, NumPy 1.19.2, PyTorch 1.7.1, XGBoost 1.4.2, scikit‐learn 0.24.1, Matplotlib 3.3.4, SciPy 1.5.2, pytorch_tabnet 3.1.1, and shap 0.39.0. The processor used was Intel**^®^** Core**™** i5‐10210U.

## 3 RESULTS

### 3.1 Summary of the best results

**FIGURE 1:**
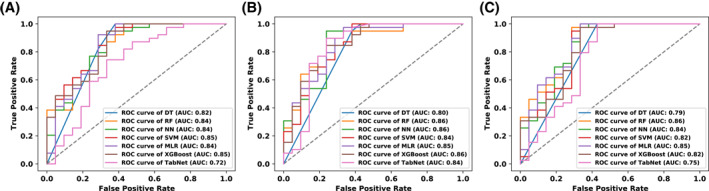
Receiver operating characteristic curves (ROC) with areas under the curve (AUC) for algorithms trained on the all features (A), best features (B), and easy‐to‐obtain features (C). Abbreviations: DT, decision tree; MLR, multiple logistic regression; NN, neural network; RF, random forest; SVM, support vector machine; XGBoost, eXtreme Gradient Boosting

**FIGURE 2:**
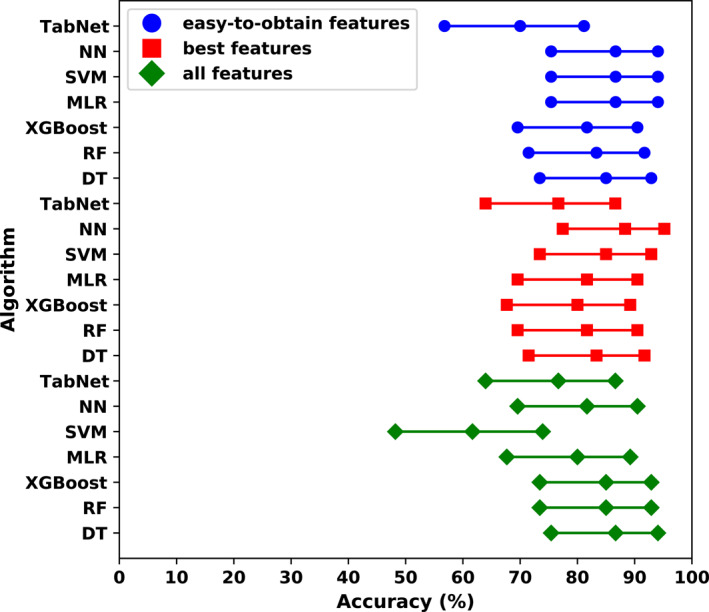
Accuracy with 95% confidence interval. Abbreviations: DT, decision tree; MLR, multiple logistic regression; NN, neural network; RF, random forest; SVM, support vector machine; XGBoost, eXtreme Gradient Boosting

The results of the best models trained on all the 3 datasets are presented in Table 3. The AUC scores are presented in Figure 1. The accuracies of each model with 95% CIs are presented in Figure 2.

**TABLE 3:** Results for all machine learning models trained on the datasets including all features, best features, and easy‐to‐obtain features

ALGORITHM	Dataset with all features					Dataset with best features					Dataset with easy‐to‐obtain features				
	ACC	DACC	SEN	SPE	AUC	ACC	DACC	SEN	SPE	AUC	ACC	DACC	SEN	SPE	AUC
**DT**	**86.67**	**64.57**	**100.00**	**61.90**	**0.82**	83.33	64.21	94.87	61.90	0.80	85.00	60.14	100.00	57.14	0.79
	**(75.41‐94.06)**	**(51.16‐76.49)**	**(90.97‐100.00)**	**(38.44‐81.89)**	**(0.69‐0.93)**	(71.48–91.71)	(50.79–76.18)	(82.68–99.37)	(38.44–81.89)	(0.69–0.9)	(73.43–92.90)	(46.68–72.57)	(90.97–100.00)	(90.97–100.00)	(0.68–0.89)
**RF**	85.00	60.14	100.00	57.14	0.83	81.67	72.53	87.18	71.43	0.86	83.33	72.71	89.74	71.43	0.86
	(73.43–92.90)	(46.68–72.57)	(90.97–100.00)	(34.02–78.18)	(0.69–0.94)	(69.56–90.48)	(59.48–83.27)	(72.57–95.70)	(47.82–88.72)	(0.75–0.95)	(71.48–91.71)	(59.67–83.41)	(75.78–97.13)	(47.82–88.72)	(0.75–0.96)
**XGBoost**	85.00	60.14	100.00	57.14	0.84	80.00	63.85	89.74	61.90	0.86	81.67	68.28	89.74	66.67	0.82
	(73.43–92.90)	(46.68–72.57)	(90.97–100.00)	(34.02–78.18)	(0.72–0.94)	(67.67–89.22)	(50.43–75.86)	(75.78–97.13)	(38.44–81.89)	(0.75–0.95)	(69.56–90.48)	(54.99–79.70)	(75.78–97.13)	(43.03–85.41)	(0.69–0.93)
**MLR**	80.00	72.35	84.62	71.43	0.83	81.67	55.53	97.44	52.38	0.85	**86.67**	**68.82**	**97.44**	**66.67**	**0.85**
	(67.67–89.22)	(59.29–83.12)	(69.47–94.14)	(47.82–88.72)	(0.70–0.95)	(69.56–90.48)	(42.13–68.37)	(86.52–99.94)	(29.78–74.29)	(0.74–0.95)	**(75.41‐94.06)**	**(55.55‐80.16)**	**(86.52‐99.94)**	**(43.03‐85.41)**	**(0.73‐0.95)**
**SVM**	61.67	74.63	53.85	76.19	0.80	85.00	64.39	97.44	61.90	0.84	**86.67**	**68.82**	**97.44**	**66.67**	**0.82**
	(48.21–73.93)	(61.73–84.98)	(37.18–69.91)	(52.83–91.78)	(0.67–0.93)	(73.43–92.90)	(50.98–76.34)	(86.52–99.94)	(38.44–81.89)	(0.73–0.94)	**(75.41‐94.06)**	**(55.55‐80.16)**	**(86.52‐99.94)**	**(43.03‐85.41)**	**(0.68‐0.95)**
**NN**	81.67	64.03	92.31	61.90	0.83	**88.33**	**77.50**	**94.87**	**76.19**	**0.86**	**86.67**	**68.82**	**97.44**	**66.67**	**0.84**
	(69.56–90.48)	(50.61–76.02)	(79.13–98.38)	(38.44–81.89)	(0.70–0.95)	**(77.43‐95.18)**	**(64.88‐87.27)**	**(82.68‐99.37)**	**(52.83‐91.78)**	**(0.74‐0.95)**	**(75.41‐94.06)**	**(55.55‐80.16)**	**(86.52‐99.94)**	**(43.03‐85.41)**	**(0.72‐0.94)**
**TabNet**	76.67	76.67	89.74	52.38	0.73	76.67	80.49	74.36	80.95	0.84	70.00	67.03	71.79	66.67	0.75
	(63.96–86.62)	(41.61–67.87)	(75.78–97.13)	(29.78–74.29)	(0.57–0.87)	(63.96–86.62)	(68.22–89.59)	(57.87–86.96)	(58.09–94.55)	(0.70–0.95)	(56.79–81.15)	(53.68–78.62)	(55.13–85.00)	(43.03–85.41)	(0.59–0.89)

*Note*: The results of the best algorithm for each dataset are marked bold. Data are presented as percentages or decimal fraction in the case of the AUC (95% confidence interval).

Abbreviations: ACC, accuracy; AUC, area under curve; DAAC, diagnostic accuracy; DT, decision tree; MLR, multiple logistic regression; NN, neural network; RF, random forest; SEN, sensitivity; SPE, specificity; SVM, support vector machine; XGBoost, eXtreme Gradient Boosting.

The best overall model was the NN trained on the dataset with the best features. It achieved an accuracy of 83.33% (6.54%–88.81%), diagnostic accuracy of 72.99% (65.31%–79.78%), sensitivity of 90.62% (82.95%–95.62%), specificity of 71.67% (58.56%–82.55%), and an AUC of 0.90 (0.84–0.95) on training data. Training results do not reflect possible effectiveness in clinical practice. They are given only as part of the overall report of a model. An accuracy of 80.14%, diagnostic accuracy of 57.86%, sensitivity of 95.79%, specificity of 55.00%, and an AUC of 0.82 were achieved during validation. These were the best validation results for the NN. After the validation process was completed, the best model was retrained on the entire training + validation dataset for final evaluation on the test set. The following results were obtained on the test set: accuracy, 88.33% (77.43%–95.18%); diagnostic accuracy, 77.50% (64.88%–87.27%); sensitivity, 94.87% (82.68%–99.37%); specificity, 76.19% (52.83%–91.78%); and AUC, 0.86 (0.74–0.95). Test set results can be used to generalize the effectiveness of a model. The best NN consisted of two hidden layers, with 12 neurons in the first layer and 129 neurons in the second layer. Rectified linear unit activation function was used in the hidden layers.

We obtained promising results for the three best models—MLR, SVM, and NN—trained on easy‐to‐obtain features. The best MLR model trained on this dataset achieved an accuracy of 79.49% (72.29%–85.53%), diagnostic accuracy of 54.83% (46.67%–62.80%), sensitivity of 96.88% (91.14%–99.35%), specificity of 51.67% (38.39%–64.77%), and an AUC of 0.83 (0.76–0.90) on training data. However, it should be noted that these results may not translate into real practice. During validation, the accuracy was 78.83%; diagnostic accuracy, 54.76%; sensitivity, 95.79%; specificity, 51.67%; and AUC, 0.78. It allowed us to retrain the model on the whole training + validation set and estimate its performance on the test set. The results for the test set were as follows: accuracy, 86.67% (75.41%–94.06%); diagnostic accuracy, 68.82% (55.55%–80.16%); sensitivity, 97.44% (86.52%–99.94%); specificity, 66.67% (43.03%–85.41%); and AUC, 0.85 (0.73–0.95).

The best SVM model trained on the dataset with easy‐to‐obtain features achieved an accuracy of 82.05% (75.11%–87.73%), diagnostic accuracy of 56.60% (48.44%–64.50%), sensitivity of 100.00% (96.23%–100.00%), specificity of 53.33% (40.00%–66.33%), and an AUC of 0.88 (0.82–0.94) on training data. During validation, the accuracy, diagnostic accuracy, sensitivity, specificity, and AUC were 80.77%, 56.45%, 97.89%, 53.33%, and 0.75, respectively. After retraining on the entire training + validation set, we obtained the following test set results: accuracy, 86.67% (75.41%–94.06%); diagnostic accuracy, 68.82% (55.55%–80.16%); sensitivity, 97.44% (86.52%–99.94%); specificity, 66.67% (43.03%–85.41%); and AUC, 0.82 (0.68–0.95). The Kernel type in this model was radial basis function.

The best NN model trained on the dataset with easy‐to‐obtain features achieved an accuracy of 82.05% (75.11%–87.73%), diagnostic accuracy of 63.99% (55.92%–71.51%), sensitivity of 94.79% (88.26%–98.29%), specificity of 61.67% (48.21%–73.93%), and an AUC of 0.86 (0.79–0.92) on training data. The validation scores were as follows: 80.77% for accuracy, 60.88% for diagnostic accuracy, 94.74% for sensitivity, 58.33% for specificity, and 0.77 for AUC. After retraining on the entire training + validation set, we achieved an accuracy of 86.67% (75.41%–94.06%), diagnostic accuracy of 68.82% (55.55%–80.16%), sensitivity of 97.44% (86.52%–99.94%), specificity of 66.67% (43.03%–85.41%), and an AUC of 0.84 (0.72–0.94) on the test set. This NN consisted of three hidden layers, with 54 neurons in the first layer, 118 neurons in the second layer, and 17 neurons in the third layer. A rectified linear unit activation function was used in the hidden layers.

### 3.2 Explainability of the best models

**FIGURE 3:**
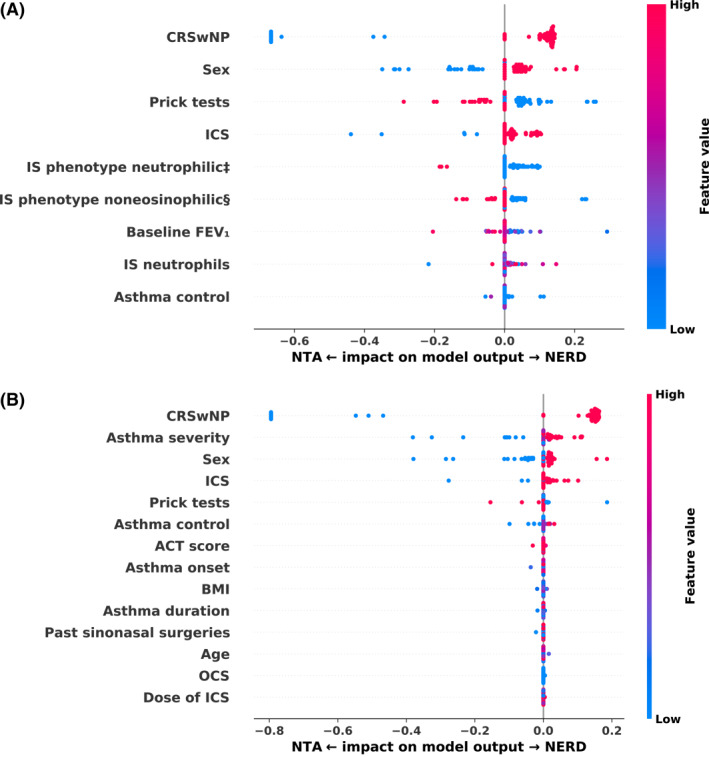
SHAP for the best model on a dataset with best features (A) and easy‐to‐obtain features (B). Abbreviations: ACT, Asthma Control Test; BMI, body mass index; CRSwNP, chronic rhinosinusitis with nasal polyposis; FEV**₁**, forced expiratory volume in the first second; ICS, inhaled corticosteroids; IS, inducted sputum; NERD, nonsteroidal anti‐inflammatory drug–exacerbated respiratory disease; NTA, nonsteroidal anti‐inflammatory drug–tolerant asthma; OCS, oral corticosteroids; SHAP, Shapley Additive exPlanations; SVM, support vector machine. ‡, § There are several accepted thresholds when defining IS phenotypes. As there is no consensus which of the threshold is the best, we included them all. ‡, threshold of 2% for eosinophiles and 60% for neutrophiles, 4 phenotypes. §, threshold of 3% for eosinophiles and 64% for neutrophiles, 3 phenotypes

“Explainability” is the concept of presenting the algorithm's prediction process in a comprehensible way. The decision‐making process of our best model can be visualized using a bee swarm plot with SHAP (Figure 3A). In this plot, features are listed from the most important at the top to the least important at the bottom. Red dots indicate a large value of a particular feature; blue dots, a small value; and violet dots, an intermediate value. Each patient is represented by a single dot on a horizontal line of a particular feature. The next step is to assess the distribution of dots: more to the left (in our case predicting towards NTA) or to the right (in our case predicting towards NERD) of the plot. The more a given dot (patient) was shifted away from the 0.0 vertical line, the stronger was the impact of that feature on that patient towards one of the classes. As an example, Figure 3A shows a large group of red dots for the CRSwNP feature on the right side of the plot. This means that the presence of CRSwNP is pushing our NN towards a conclusion that patients with this condition have NERD. A similar visualization was done for the SVM trained on a set of easy‐to‐obtain features (Figure 3B).

## 4 DISCUSSION

The COVID‐19 pandemic has greatly affected our daily practice. The use of aerosol‐generating procedures such as OAC, spirometry, and IS testing has been restricted due to the risk of virus transmission. As the pandemic was not likely to stop, the authors of guidelines focused on how to safely perform some of these procedures,^17^ and an urgent need emerged to develop tools for the diagnosis of aspirin hypersensitivity based on clinical data that are easy to obtain during the pandemic. Recently, it was shown that an informatics algorithm based on electronic health records datasets including inflammatory biomarkers, could successfully identify, with a high positive predictive value, both known and previously undiagnosed cases of NERD.^18^ ML techniques are increasingly used in medicine, including in the fields of allergology and pulmonology. Examples include personalized systems that predict asthma exacerbations^19^ or survivability estimators in lung cancer.^20^ Our previous study showed promising results for an artificial NN in terms of discriminating between NERD and NTA.^7^ However, the features input into the ML model comprised multiple data that were obtained not only from medical history but also included clinical and laboratory parameters (such as IS inflammatory biomarkers). In this study, we investigated whether in the pandemic setting such a diagnosis could be made only on the basis of easy‐to‐obtain clinical features and data from medical history, excluding procedures generating infectious aerosol.

While there are numerous ML techniques, it is difficult to predict which algorithm will work best for a particular problem. Usually, algorithms have to be compared before the best one is selected. In our study, we attempted to create a classifier that would be able to distinguish between patients with NERD and NTA using only clinical and laboratory data. In our study, we decided to assess and compare 7 ML techniques for obtaining the best results. The NN trained on the set of best features was shown to provide the best results. These data appeared to be most useful in discriminating between NERD and NTA. Surprisingly, however, the set contained inflammatory phenotypes based on sputum induction, which is difficult to perform during the pandemic.

The NN trained on the best features set required the following input data: baseline FEV₁, percentage of IS neutrophils, sex, presence of CRSwNP, ICS use, skin prick test results, level of asthma control, and information about IS phenotypes. The algorithm obtained a sensitivity of 95% and a specificity of 76%, as compared with a sensitivity of 89% and a specificity of 93% for OAC. Thus, in terms of sensitivity, our network outperforms aspirin challenge, but it has worse specificity. The use of NN has the following advantages over OAC: no risk of anaphylaxis, no need of well‐trained medical team or hospitalization (which is particularly important during the pandemic), and cost effectiveness.

The three promising models trained on easy‐to‐obtain features, namely, MLR, SVM, and NN, required the following input data: age, sex, body mass index, age at asthma onset, asthma duration, ACT score along with asthma control and severity levels, history of sinonasal surgery, information on ICS and OCS use (with dosage), skin prick test results, and the presence of CRSwNP. Interestingly, those algorithms obtained a sensitivity of 97% and a specificity of 67%. Their additional advantage is no need for laboratory testing. Despite their relatively low specificity, these models could serve as valuable screening tools owing to easily accessible input data and high sensitivity.

Our study has several limitations. The patient population was homogenous—all patients were White and came from a single country. Moreover, they all had stable disease and all of them had a FEV_**1**_ > 70% of predicted value. There is some possibility that, in real practice, patients with NTA may be classified as hypersensitive to aspirin by the ML model. In doubtful cases, OAC should be performed, which means that patients might need to wait until the use of OAC is safe.

## 5 CONCLUSIONS

The COVID‐19 pandemic has greatly restrained our diagnostic possibilities by placing limitations on the use of procedures linked to aerosol release, such as lung function tests, IS testing, and spirometry‐based provocation tests. To our knowledge, we are the first to compare several ML techniques in terms of their ability to differentiate between NERD and NTA, including models trained only on easy‐to‐obtain clinical features. Our study revealed potentially the most efficient techniques, including the NN, SVM, and MLR.

Although OAC remains the gold standard for the diagnosis of aspirin hypersensitivity, the use of ML could facilitate patient care by reducing delays in diagnosis and improving safety, especially during the pandemic. ML techniques are easy to use, safe, and offer very good results, thus becoming a very promising option in the diagnosis of NERD. However, before these techniques become routinely used in patients with asthma, our findings need to be externally validated on populations worldwide, and, ideally, confirmed in large cohort studies.

## AUTHOR CONTRIBUTIONS

**Study idea**: Agnieszka Gawlewicz‐Mroczka, Adam Pytlewski, Adam Ćmiel, Lucyna Mastalerz. **Medical expertise**: Agnieszka Gawlewicz‐Mroczka, Natalia Celejewska‐Wójcik, Anna Gielicz, Marek Sanak, Lucyna Mastalerz. **Provision of the data base**: Adam Ćmiel, Lucyna Mastalerz. **Statistical analysis**: Adam Pytlewski, Adam Ćmiel, Lucyna Mastalerz. **Machine learning deployment**: Adam Pytlewski, Adam Ćmiel. **Manuscript writing**: All authors. **Final approval**: All authors.

## CONFLICT OF INTEREST

The authors declare that they have no competing interests.

## Supporting information

Supporting Information S1

Supporting Information S2

## Data Availability

The data that support the findings of this study are available from the corresponding author upon reasonable request.
