# Supplementary material for: Machine learning in the diagnosis of asthma phenotypes during coronavirus disease 2019 pandemic
Source: Clin Transl Allergy. 2022 Oct 19;12(10):e12201. doi: 10.1002/clt2.12201 (PMC9579891; doi:10.1002/clt2.12201)
Supplement: Supplementary file 1 — Supporting Information S1 [file CLT2-12-e12201-s002.docx]

**Supplementary materials**

Contents

1. FigureS1*.* Study flowchart.
2. Methods: Algorithms

**Algorithms**

**Decision tree**

A DT consists of the root node, internal nodes, and leaf nodes. The root contains all patients. Next, the internal nodes divide patients into smaller groups based on a particular feature. Finally, there are the leaf nodes. Each leaf should contain only patients with NERD or NTA. At each step, the DT is looking for the most profitable way of split.

**Random forest**

An RF is an ensemble of many DTs trained separately. The more DTs are used to create an RF, the more complex it becomes. Individual DTs in the RF are usually trained on bootstraps of the entire dataset and only on part of the features. When the RF makes prediction, all of its DTs vote. An option with more votes wins and becomes a prediction of the RF.

**eXtreme Gradient Boosting**

Like RFs, XGBoost trees consist of many DTs. However, they are trained in a different manner. In XGBoost, DTs are grown one after another and each one is trying to improve mistakes done by its predecessor.

**Multiple logistic regression**

MLR is a linear model that can take multiple features and use them in a sigmoid function to output the probability of the output.

**Support vector machines**

SVMs can efficiently perform a non-linear classification using what is called the kernel trick, implicitly mapping their inputs into high- or infinite-dimensional feature spaces. A linear decision surface is constructed in this new feature space. To keep the computational load reasonable, the mappings used by SVM schemes are designed to ensure that dot products of the pairs of input data vectors may be computed easily in terms of the variables in the original space, by defining them in terms of a kernel function selected to suit the problem. The advantage of this approach is absence of local minima in the SVM optimization problem.

**Neural networks**

NNs are nonlinear complexed functions used for various tasks. Dense NNs can be used to solve tasks on tabular data and these networks has been used in our project. These NNs are called ‘dense’ because each neuron from the previous layer is connected with other neuron in the next layer. The NN comprises of an input layer, hidden layers, and an output layer (which predicts the output).

**TabNet**

TabNet is a new deep NN architecture proposed in 2019. Its main advantages described by the authors include end-to-end learning (a tabular dataset does not have to be pre-processed) as well as the use of sequential attention, which enables soft feature selection and interpretability. The authors showed that TabNet can outperform many state-of-the-art algorithms in various classification tasks. TabNet models in our research were first pre-trained during a self-supervised learning process. It relied on masking part of input features and a model learning to predict them with the rest of unmasked input data. The idea was to improve the performance of the model when dealing with a small dataset.

**Figures titles and legends**

**Figure S1.** Study flowchart.

Abbreviations: DT – decision tree; MLR – multiple logistic regression; NN – neural network; train+val – training+validation; RF – random forest; SVM – support vector machine; XGBoost – eXtreme Gradient Boosting; CV – cross validation.
