# Supplementary material for: Machine learning in the diagnosis of asthma phenotypes during coronavirus disease 2019 pandemic
Source: Clin Transl Allergy. 2022 Oct 19;12(10):e12201. doi: 10.1002/clt2.12201 (PMC9579891; doi:10.1002/clt2.12201)
Supplement: Supplementary file 2 — Supporting Information S2 [file CLT2-12-e12201-s001.pdf]

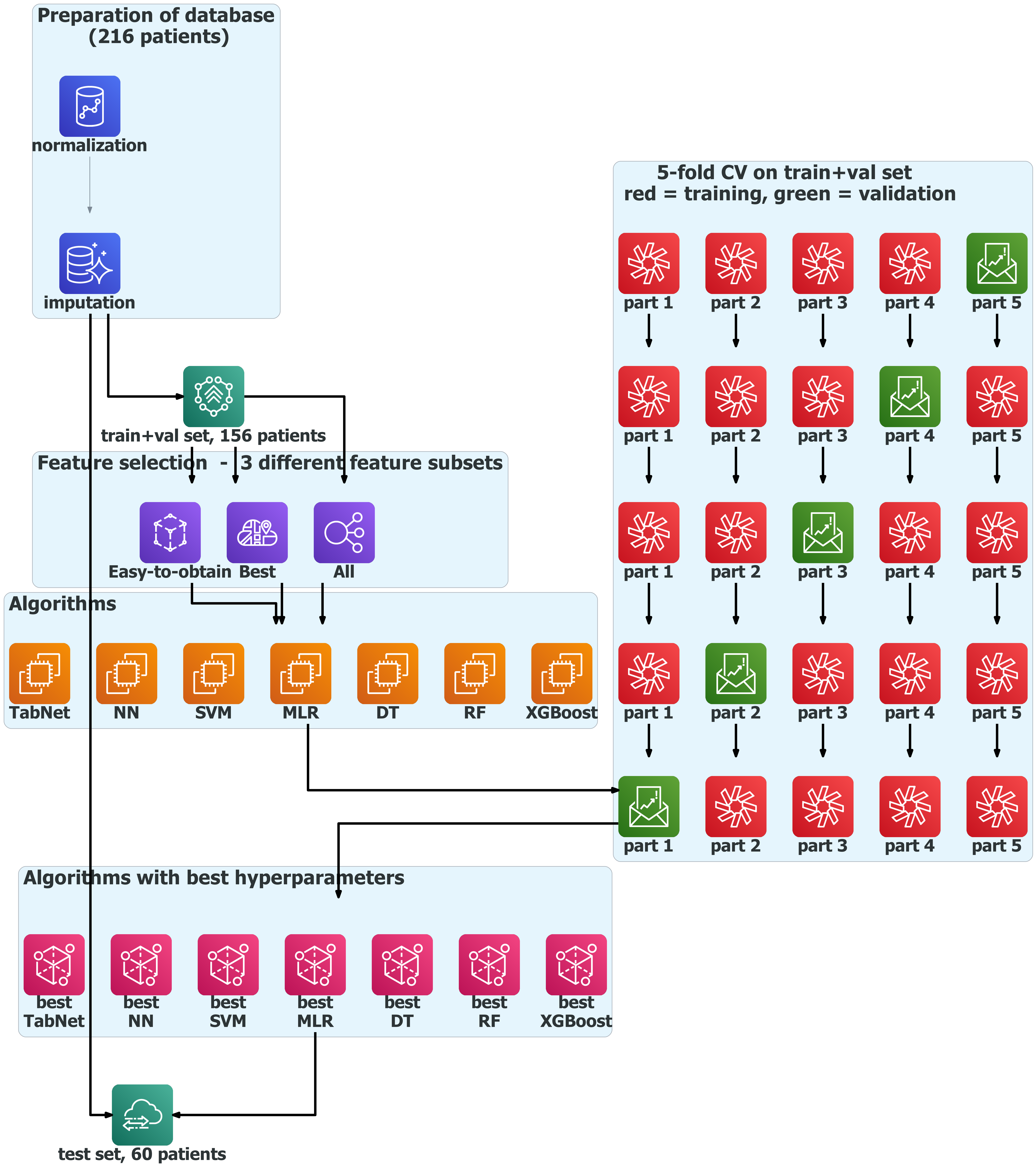
Preparation of database (216 patients)
normalization
imputation
train+val set, 156 patients
Feature selection - 3 different feature subsets
Easy-to-obtain
Best
All
Algorithms
TabNet
NN
SVM
MLR
DT
RF
XGBoost
5-fold CV on train+val set
red = training, green = validation
part 1
part 2
part 3
part 4
part 5
Algorithms with best hyperparameters
best TabNet
best NN
best SVM
best MLR
best DT
best RF
best XGBoost
test set, 60 patients
